# Complete mitochondrial genome of the spotted nutcracker *Nucifraga caryocatactes* (Passeriformes: Corvidae) from Shan’xi Province, China

**DOI:** 10.1080/23802359.2020.1778565

**Published:** 2020-06-16

**Authors:** Dehuai Meng, Zhirong Zhang, Zongzhi Li, Yuhui Si, Qiuxia Guo, Zhensheng Liu, Liwei Teng

**Affiliations:** a College of Wildlife and Protected Area, Northeast Forestry University, Harbin, P. R. China; b State-Owned Forest in Taiyuan, Taiyuan, P. R. China; c Key Laboratory of Conservation Biology, National Forestry and Grassland Administration, Harbin, P. R. China

**Keywords:** Complete mitochondrial genome, *Nucifraga caryocatactes*, Corvidae

## Abstract

We determined the whole mtDNA genome of the Spotted Nutcracker (*Nucifraga caryocatactes*) in Tianlong Mountain, Shan’xi Province, China. The complete mitochondrial genome is 16,914 bp in length and consists of 13 protein-coding genes (PCGS), 22 *tRNA* genes, 2 *rRNA* genes, and 1 control region (D-loops). The nucleotide composition is 25.08% A, 25.08% T, 24.75% G, and 25.08% C. The result of phylogenetic analysis showed that there was close genetic relationship between *N. caryocatactes* and *N. columbiana*. It is expected that the complete mitochondrial genome presented here will contribute to the analysis of species distribution.

The spotted nutcracker (*Nucifraga caryocatactes*) is a widespread resident species the Palearctic from Central Europe to Asia (Surhone et al. 2010; Dohms and Burg 2014). *N*. *caryocatactes* is a bird slightly larger than the Eurasian jay (*Garrulus glandarius*), with a much larger beak and a skinny-looking head without a crown. Its body feathers are mostly chocolate brown with distinct white spots and stripes, and its wings and upper tail are actually black with a blue–green sheen. It is one of two species of nutcracker, the other being the Clark’s Nutcracker (*Nucifraga columbiana*), which is replacing in western North America (Surhone et al. 2010; Miller et al. 2010).

We sequenced the mitochondrial genome of *N. caryocatactes*. Our samples were obtained from fresh muscle of the *N. caryocatactes* from natural death in the Tianlong Mountain, Shan’xi Province, China (112°20′45.23′′E, 37°43′00.95′′N). These specimens were stored in College of Wildlife and Protected Area, Northeast Forestry University (No. XY201911).

The complete mitochondrial genome of *N. caryocatactes* (GenBank: MT506195) was 16,914 bp in length and consisted of 37 genes, including13 protein-coding genes (PCGS), 2 *rRNA* genes (12S *rRNA* and 16S *rRNA*), 22 *tRNA* genes, and 1 control region (D-loop). The nucleotide composition is 25.08% A, 25.08% T, 24.75% G, and 25.08% C. The total length of 13 PCGS is 11,418 bp in length, all of which are encoded on the same strand except for ND6 in the heavy strand (H strand). In 13 PCGS, except COX1 begins with GTG, CYTB begins with ATC, and ND6 begins with TTA, the remaining 10 PCGS begin with ATG (*ND1, ND2, COX2, ATP8, ATP6, COX3, ND3, ND4L, ND4*, and *ND5*) as start codon. The total length of 22 *tRNA* genes is 1423 bp in length, and ranges from 67 to 76 bp are interspersed along the whole genome. The sequence length of the 12 s RNA and 16 s RNA is 979 and 1604 bp, and D-loop regions (control regions) are 1227 bp.

The phylogenetic relationship was inferred by using the maximum likelihood method based on the Tamura–Nei model (Tamura and Nei 1993) and conducted in MEGA7 (Kumar et al. 2016). The bootstrap consensus tree inferred from 1000 replicates is taken to represent the evolutionary history of the taxa analyzed (Felsenstein 1985). The phylogenetic tree appeared that the phylogenetic relationship of *N. caryocatactes* is very close to *N. columbiana* (Figure 1).

**Figure 1.**
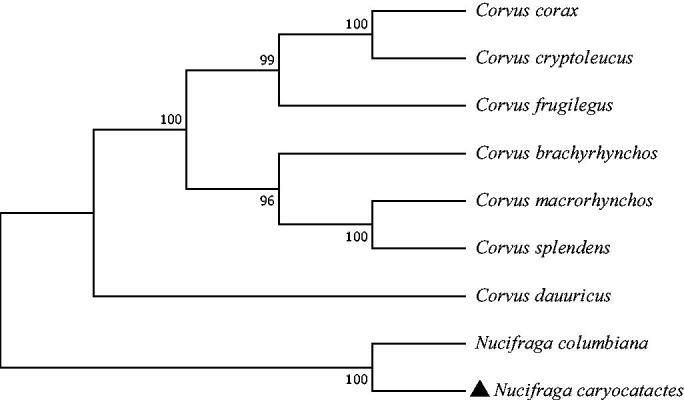
Phylogenetic tree generated using the maximum likelihood method based on complete mitochondrial genomes of nine species in Passeriformes: Corvidae. GenBank accession numbers: *Corvus corax* (KX245137.1), *Corvus cryptoleucus* (NC_034839.1), *Corvus frugilegus* (Y18522.2), *Corvus brachyrhynchos* (KP403809.1), *Corvus macrorhynchos* (MN069302.1), *Corvus splendens* (KP019937.1), *Corvus dauuricus* (NC_046029.1), *Nucifraga columbiana* (KF509923.1), and *Nucifraga caryocatactes* (This study).

## Data Availability

The data that support the findings of this study are openly available in GenBank of NCBI at https://www.ncbi.nlm.nih.gov/, reference number MT506195.
